# Characteristics of disease progression and genetic correlation in ambulatory Iranian boys with Duchenne muscular dystrophy

**DOI:** 10.1186/s12883-022-02687-1

**Published:** 2022-05-02

**Authors:** Gholamreza Zamani, Sareh Hosseinpour, Mahmoud Reza Ashrafi, Mahmoud Mohammadi, Reza Shervin Badv, Ali Reza Tavasoli, Masood Ghahvechi Akbari, Ali Hosseini Bereshneh, Reza Azizi Malamiri, Morteza Heidari

**Affiliations:** 1 Pediatrics Center of Excellence, Department of Pediatric Neurology, Children’s Medical Center, Tehran University of Medical Sciences, Tehran, Iran; 2 Department of Pediatric Neurology, Vali-e-Asr Hospital, Imam Khomeini Hospital Complex, Tehran University of Medical Sciences, Tehran, Iran; 3 Physical Medicine and Rehabilitation Department, Children’s Medical Center , Tehran University of Medical Sciences, Tehran, Iran; 4 Prenatal Diagnosis and Genetic Research Center, Dastgheib Hospital, Shiraz University of Medical Sciences, Shiraz, Iran; 5 Department of Pediatric Neurology, Golestan Medical, Educational, and Research Center, Ahvaz Jundishapur University of Medical Sciences, Ahvaz, Iran

**Keywords:** Duchenne muscular dystrophy, Phenotype and genetic correlation, Iranian patients

## Abstract

**Background:**

Duchenne muscular dystrophy (DMD) is the most common muscular dystrophy in the pediatric population. The manifestations of this disease include progressive muscle weakness, gait dysfunction, and motor impairment, leading to a loss of ambulation by the age of 13 years. Molecular diagnosis is the standard diagnostic tool for DMD. This study aimed to investigate disease progression and genetic patterns in Iranian ambulant boys and to find the correlation between genotypes and motor function phenotypes.

**Methods:**

This study was performed on 152 DMD patients. Clinical history, including the disease phenotype, steroid therapy, and the North Star Ambulatory Assessment (NSAA) score, was taken for all the patients. Molecular diagnoses were confirmed by multiplex ligation-dependent probe amplification and next-generation sequencing tests.

**Results:**

A total of 152 Iranian DMD patients were examined in this study. The mean age at the time of disease onset was 4.04 ± 2.00 years, and the mean age at diagnosis was 5.05 ± 2.08 years. The mean age of ambulation loss was 10.9 years. Contracture was reported in 38.9% of cases. In terms of age, the mean total NSAA score showed a peak at 4 years of age, with a mean NSAA score of 24. Annual changes in the NSAA score were determined for all cases, based on the mutation type and exon site. Deletion mutation was found in 79.1% of cases, duplication in 6.8%, nonsense in 12.8%, and splice site in 1.4%. The most common single exon deletion was exon 44 (5.3%), and the most common multiexon deletions were attributed to exons 45–50 and exons 45–52 (4.6%). The results did not indicate any correlation between the mutation type and age at the time of disease onset, loss of ambulation age, and wheelchair dependence; however, a significant association was found between contracture and mutation type. The results showed a significant difference in the NSAA score between the deletion and nonsense groups at the age of 3 years (*P* = 0.04). No significant correlation was found between the phenotype and exon site. Overall, 91.1% of the study population had a history of corticosteroid use, and 54.1% showed compliance with rehabilitation therapy.

**Conclusion:**

This study demonstrated the phenotypes and mutational features of Iranian DMD boys and provided information regarding the natural motor history of the disease, disease progression, diagnosis, and status of DMD management in Iran. The present findings can promote the development of clinical trials and future advanced molecular therapies in Iran.

**Supplementary Information:**

The online version contains supplementary material available at 10.1186/s12883-022-02687-1.

## Background

Duchenne muscular dystrophy (DMD) is the most common muscular dystrophy in the pediatric population, affecting one in every 5000 live male births [1]. It is defined as a recessive, X-linked neuromuscular disorder [26], caused by mutations in the *DMD* gene as the largest human gene located on chromosome Xp21. Depending on the mutation site, the production of full-length dystrophin or other dystrophins is terminated in various tissues in DMD [21]. Especially in the striated muscle, the loss of full-length dystrophin results in the deficient assembly of dystrophin-dystroglycan protein complex, which contributes to the stabilization of sarcolemma during muscle contraction, relaxation, or stretch [27].

The first manifestations of DMD include progressive muscle weakness, gait dysfunction, and motor impairment. The symptoms typically emerge between the ages of three and 5 years. The progression of muscle pathology leads to the continuous progression of muscle weakness, resulting in the loss of ambulation (LoA) and wheelchair dependence by the age of 13 years. Other manifestations include a decline in cardiac function and breathing problems; aggravation of these two systems is the most serious cause of death in the second and third decades of life among DMD patients [24].

Histopathology was previously used as the first-line diagnostic tool for DMD. However, it was replaced by non-invasive molecular tests, which can define the type of mutation in the *DMD* gene. This method defines the type of mutations in the *DMD* gene, including deletions (57–65%), duplications (6–11%), and point mutations [9, 15, 26, 29]. The multiplex ligation-dependent probe amplification (MLPA) is the first molecular test, which was used as a standard diagnostic tool for mutation analysis [4, 5, 23]. The MLPA can detect exonic deletions and duplications in 79 exons of *DMD* gene, although it misses point mutations, such as nonsense, missense, small insertions, and splice sites. Sequencing is the second step in molecular studies to evaluate point mutations (25–30% of molecular pathology) in the *DMD* gene. Today, the more efficient method of next-generation sequencing (NGS) is used rather than the time-consuming Sanger sequencing method [11, 18, 20, 27].

Generally, any mutation that disrupts the reading frame of dystrophin or induces a premature stop codon results in the loss of functional dystrophin and causes severe DMD, whereas mutations that maintain the dystrophin reading frame typically result in a milder phenotype, known as Becker muscular dystrophy (BMD) [2, 7, 16, 22]. Therefore, establishing the link between the genetic patterns and clinical phenotypes of DMD in the pediatric DMD population can help us determine the mutation-specific nature and clinical behavior of this disease and may be valuable for improving the patients’ survival, mortality, and life expectancy. Besides, it is crucial to increase our knowledge of specific mutations in DMD to identify patients eligible for new treatments. It is also important to pay particular attention to phenotype variations in disease progression for delivering standard care (glucocorticoid and rehabilitation). Our understanding of these variations can make significant changes in the natural history of DMD and may lead to the application of recently approved therapeutic methods, which address specific mutations (skipping exons 45, 51, and 53 and read-through nonsense mutations).

Dystrophin is a rod-shaped protein, consisting of four domains. The actin-binding domain spans from exon 2 to exon 8 (amino acids 14–240); the central rod domain spans from exon 8 to exon 61 (amino acids 253–3040) and is the largest domain of dystrophin, containing 24 spectrin-like triple-helical elements; the cysteine-rich domain spans from exon 63 to exon 69 (amino acids 3080–3360); and the carboxy-terminal domain spans from exon 70 to exon 79 (amino acids 3361–3685). Generally, the position and type of mutations have different consequences. In-frame deletions cause slight changes in the structure of protein and are associated with the milder form of macular dystrophy, whereas frameshift deletions and/or nonsense mutations may disrupt the structure of protein or nonsense-mediated mRNA decay (NMD), associated with the severe form of DMD [1, 17, 25].

With this background in mind, the present study aimed to describe disease progression and genetic patterns in the Iranian pediatric DMD population and to find the correlation between genotypes and motor function phenotypes of DMD.

## Materials and methods

### Patients

This study was performed on 152 DMD patients, referred to the neuromuscular clinic of Children’s Medical Center (CMC) and the Iranian Muscular Dystrophy Association over the last 5 years. This study was approved by the Ethics Committee and Institutional Review Board (IRB) of Tehran University of Medical Sciences (IRB code: IR.TUMS.CHMC.REC.1398.026), and informed consent was obtained from the child’s caregiver if needed.

Before statistical analyses, DMD was defined based on both *DMD* gene mutation and DMD phenotype. The inclusion criteria were available clinical and genetic data and no history of other systemic or neurological disorders. The patients’ medical records were collected, and the patients’ parents were interviewed to complete the information. Data regarding the patients’ clinical history and DMD phenotype, including the patient’s age at the time of disease onset, age at the time of genetically confirmed diagnosis, motor delay, LoA age (LoA was defined as inability to perform the 10-m walk test independently, associated with permanent wheelchair use and confirmed by a pediatric neurologist), wheelchair dependence, presence of calf muscle hypertrophy and contracture, and steroid therapy (prednisolone or deflazacort), were collected. The Iranome database (http://www.iranome.ir/gene/ENSG00000198947) was also used to double-check the novelty of detected variants and/or mutations in the Iranian population.

### North star ambulatory assessment (NSAA)

In this study, the North Star Ambulatory Assessment (NSAA) was used to determine the total score of motor function. The NSAA was used to represent an important aspect of DMD phenotypes and to evaluate the progression of weakness at the age of 3 years, 3 years and 6 months, and 4 years until 12 years annually. Generally, it is a validated unidimensional functional scale for ambulant DMD boys in clinical practice. It consists of several items, evaluating the necessary skills to keep the patient functionally ambulant. There are eight items in this scale that are appropriate for the age of 3 years; there are 13 items that are appropriate for the age of 3 years and 6 months; and all 17 items are appropriate for the age of 4 years. The total score is determined by summing the scores of all individual items. The scores range from 0 (“when all activities are failed”) to 34 (“if all activities are achieved”). All medical records of patients were collected during the study (5 years). The NSAA scores of all patients were assessed by a pediatric neurologist and reported orderly in the patient’s medical record according to the clinic policy from the first visit until the final visit. All longitudinal NSAA data are now available.

### Molecular genetic testing

Molecular diagnoses were established using the MLPA and NGS tests. The MLPA is the most reliable test to identify deleted or duplicated exons accurately. The distribution of mutation sites in 79 DMD exons was classified as proximal or distal to exon 45.

### MLPA

The MLPA test was used to identify potential deletions and duplications in all 79 exons of *DMD* gene, located on Xp21.2 locus, using SALSA MLPA Probemix P034-P035 Kit (MRC Holland, Amsterdam, Netherlands). This kit contains probes for all 79 exons of *DMD* gene, as well as one probe for the exon 1 DP427c alternative of DMD. The probes were divided into two probemixes of P034 and P035, with the potential to detect deletions and duplications in one or more segments of *DMD* gene. Each probemix contains 45 different probes that produce amplicons, ranging from 129 to 490 nucleotides; it also includes 10 controls that generate products shorter than 120 nucleotides. Patients with negative MLPA results were analyzed by NGS.

### NGS and sanger sequencing

All coding sequences, as well as highly conserved exon-intron splice sites of *DMD* gene, were analyzed by polymerase chain reaction (PCR) and sequencing of both DNA strands. The amplicons of *DMD* gene, containing exons and highly conserved exon-intron splice sites, were generated and then analyzed by amplicon-based NGS, using an Illumina HiSeq 4000 platform. The average coverage of all amplicons was more than 20X. The regions that were not covered by NGS, as well as regions with poor quality, were re-sequenced by Sanger sequencing to achieve a coverage of 100%. Moreover, an in-house pipeline, including Burrows-Wheeler Aligner software (version 0.7.15-r1140), was utilized to align the sequence reads to the reference genome (GRCh37.p10).

Mark duplication and base quality recalibration of aligned reads were performed using the GATK MarkDuplicate and BaseRecalibrator tools, respectively. The Genome Analysis Toolkit (GATK) HaplotypeCaller, FreeBayes, and Samtools variant calling tools were also used for calling single nucleotide and indel variants. Coding exons alongside +/− 20 flanking sequences of introns were covered. Next, all pathogenic and likely pathogenic variants in DMD were investigated. Finally, Sanger sequencing was performed in both directions (forward and reverse) to confirm the detected variants, using Applied Biosystems 3130 Genetic Analyzer.

### Statistical analysis

Demographic data and clinical characteristics were measured using statistical tests and then subjected to descriptive analysis between the groups. Descriptive statistics, including mean ± SD, were used to represent quantitative variables, and frequency and percentage were measured for qualitative variables. ANOVA test was used to analyze the relationship between quantitative variables, including the NSAA score, disease onset age, and diagnosis age, and qualitative variables, including the mutation type. Moreover, t-test was performed to analyze the relationship between quantitative variables, including the NSAA score, disease onset age, and diagnosis age, and qualitative variables, including the exon site. Besides, Chi-square test was performed to analyze the relationship between qualitative variables (contracture, motor delay, and LoA) and qualitative variables (mutation type and exon site). Comparison of changes in the NSAA score over time between the mutation types was also performed using repeated measures ANOVA test, and Bonferroni post-hoc test was utilized to analyze the distance between mutations at different time points. The level of statistical significance was set at *P* < 0.05.

## Results

### Demographic data

This study was performed on 152 Iranian boys with a genetically approved diagnosis of DMD, referred to Children’s Medical Center. The patients’ motor function and ambulatory status, as well as the genetic profile, were investigated. The mean age of the patients at disease onset was 4.04 ± 2.00 years, and the mean age at diagnosis was 5.05 ± 2.08 years. A history of motor delay was reported in 47.7% of cases, calf hypertrophy was reported in 97.1% of cases, and progressive weakness was reported in 86.2% of cases by the families.

### Ambulation characteristics

**Fig. 1:**
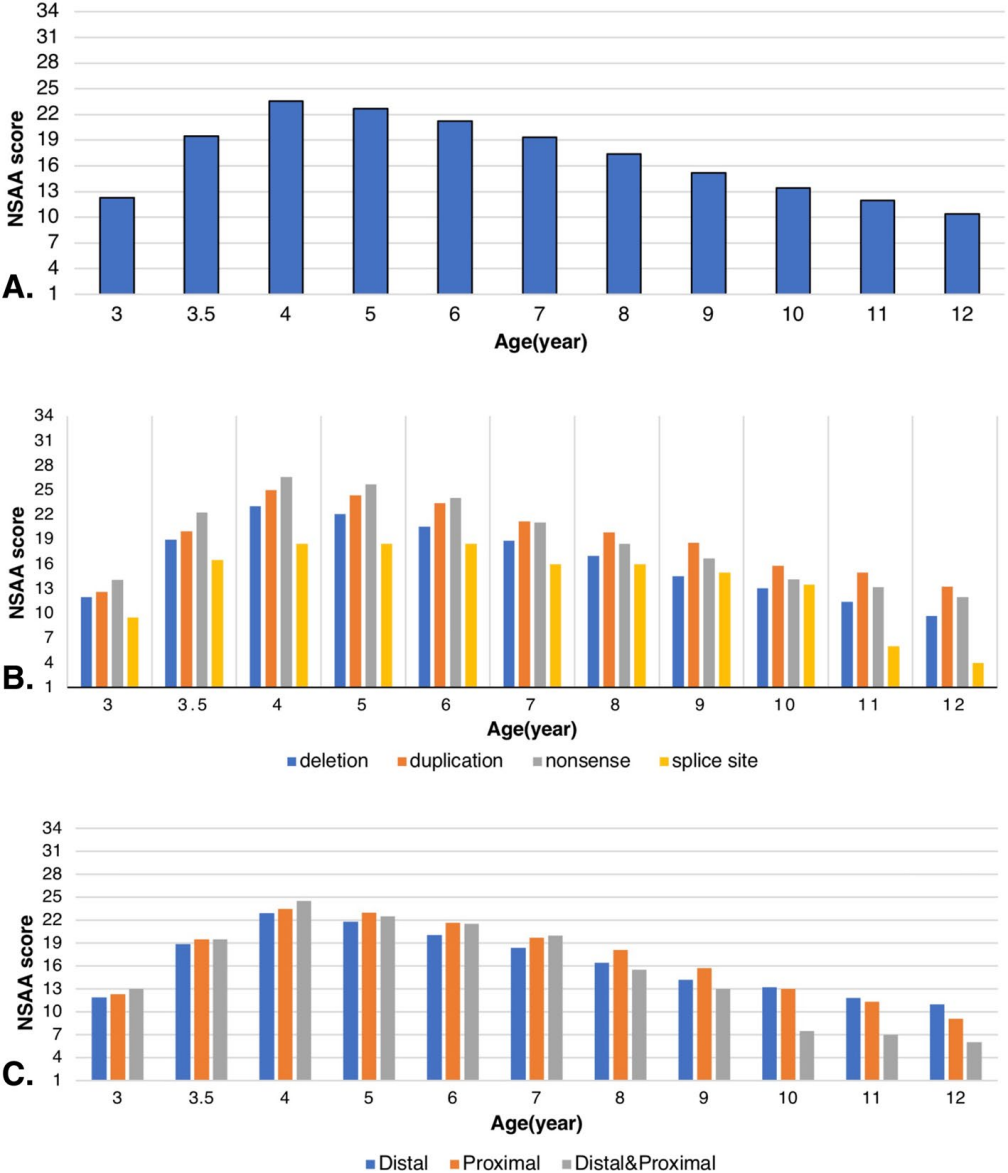
Yearly changes in the NSAA score in all DMD boys (**A**), NSAA score based on “mutation” type groups (Deletion; *n* = 120, Duplication; *n* = 11, Nonsense; *n* = 19, Splice site; *n* = 2) (**B**), and “mutation site groups”(**C**)

In this study, 65 (42.7%) patients were dependent on a wheelchair, and the mean age of LoA and wheelchair dependence was 10.9 years. Contracture was detected in 38.9% of cases, mostly in the ankles and knees (78 and 33%, respectively). A total of 1345 NSAA assessments were available for analysis. Table 1 presents the number of DMD patients according to the number of NSAA score measurements. The duration of follow-up ranged from almost 1 year to 12 years. The mean total NSAA score versus age peaked at 4 years, with a mean NSAA score of 24. Following the peak, there was a decline in the NSAA score. Annual changes in the NSAA score were studied in all patients with DMD and categorized based on the mutation type and exon site. The rate of decline in the NSAA score was 1.67 units/year for all patients (Fig. 1A). The NSAA score was significantly different between ambulant and wheelchair-bound cases after 4 years, with higher NSAA scores in ambulant boys as opposed to lower NSAA scores in wheelchair-dependent cases.

**Table 1:** The number of DMD patients according to the number of NSAA score measurement

The number of DMD patients according to the number of NSAA score measurement											
**Total measurement: 1345**											
**Number of Measurement**	1 time	2 times	3 times	4 times	5 times	6 times	7 times	8 times	9 times	10 times	11 times
**Number of patients**	–	3 p	4 p	9 p	7 p	7 p	14 p	14 p	9 p	12 p	73 p

*P* patients, *DMD* Duchenne muscular dystrophy, *NSAA* North Star Ambulatory Assessment

### Genetic profile

Based on the analysis of genetic profile in DMD patients, the diagnosis method was MLPA in 82.9% of cases (*n* = 126), while NGS was used for boys who were clinically suspected of DMD, but had negative MLPA results. NGS was the diagnostic tool in 17.1% of cases (*n* = 26). Regarding the type of mutation, deletion mutations were found in 79.1% of cases, duplication was found in 6.8% of cases, nonsense was found in 12.8% of cases, and splice site was found in 1.3% of cases. The most common single exon deletion was exon 44 deletion (5.3%), and the most common multiexon deletions were 45–50 and 45–52 exons (4.6% for both). Regarding the distribution of mutation sites in 79 DMD exons, 47.4% of all mutations occurred in the distal part, 35.5% occurred in the proximal part, and 1.3% occurred in both distal and proximal sites.

Among patients with deletion mutations approved by MLPA, 57% (*n* = 72/126) were located in the distal part; the largest deletion was the deletion of 3–44 exons in the proximal part. The variants, pathogenicity, and number of reports for each variant were categorized according to the Global Variome shared LOVD (Supplementary Table S1). Deletions and duplications were reported in the Iranome database. Overall, 85.8% of diagnoses were confirmed by MLPA, with sensitivity of 63 to 79.5% according to previous studies. The deletion rate was 93% in MLPA-confirmed DMD cases. Deletions were the most prevalent mutation, accounting for 79.1% of all mutations, followed by nonsense mutations, duplications, and splice sites (12.8, 6.8, and 1.3%, respectively).

### Genotype-phenotype correlation

The correlation between the mutation type and phenotype was assessed in this study. The results did not show any significant correlation between the mutation type and age at the time of disease onset, LoA age, and wheelchair dependence. The LoA was analyzed as a time-to-event variable, using Kaplan-Meier test, and log-rank test was used to estimate the effect of mutation type on the LoA age and wheelchair dependence (log-rank: Chi-square = 2.043, df = 3, *P* = 0.564). Similar analyses were performed to examine the effect of mutation site (log-rank: Chi-square = 0.794, df = 2, *P* = 0.672). The results showed that mutation class and site did not affect the LoA time.

A significant association was found between contractures and mutation type (*P* = 0.03). The presence of contracture was seen in 55/152 DMD patients, including 11/19 cases with nonsense mutations (57.9%), 55/120 cases with deletion mutations (45.9%), 2/10 cases with duplication mutations (20%), and 2/2 cases with splice site mutations as the smallest group. It occurred more often in patients carrying nonsense mutations compared to patients with other mutations. Based on the repeated measure test, the slope of changes from three to 12 years represented no significant difference between the four mutation types (deletion, duplication, nonsense, and splice site) and also the exon sites (distal/proximal) (*P* = 0.8) (Fig. 1).

There was no significant difference between the mutation type and the NSAA score at the onset of disease progression after 4 years. Based on the subgroup analysis using post-hoc test, a significant difference was found in the NSAA score between the deletion and nonsense groups at the age of 3 years (*P* = 0.04) (Fig. 1B, Table 2). The rate of decline in the NSAA score was 1.65 units/year in the deletion mutation group, 1.86 units/year in the nonsense mutation group, and 1.25 units/year in the duplication mutation group. Moreover, the genotype-phenotype correlation was assessed by evaluating the relationship between the mutation sites and phenotype. There was no significant correlation between the phenotypes and exon sites. The LoA age was 10.7 ± 3.10 and 11.02 ± 2.09 years in DMD boys with affected exons in the distal and proximal parts, respectively. The mean age at the time of disease onset was 4.05 ± 1.80 and 3.6 ± 2.2 years in patients with distal and proximal part involvements, respectively. Wheelchair dependence was reported in 45.6% of cases with distal exon mutations and 38.5% of cases with proximal exon mutations. There was no significant correlation between motor delay and genotypes. Similarly, no significant correlation was observed between the mutation subgroups and phenotypes (Supplementary Table S2).

**Table 2:** NSAA score (Mean ± SD) in mutation type groups (deletion, duplication, nonsense and splice site) at each time point

Age (year)	Deletion (***n*** = 120) (Mean ± SD)	Duplication (***n*** = 11) (Mean ± SD)	Nonsense (***n*** = 19) (Mean ± SD)	Splice site (***n*** = 2) (Mean ± SD)
3	11.97 ± 3.23	12.6 ± 3.6	14.11 ± 2.35	9.5 ± 3.54
	*n* = 120	*n* = 10	*n* = 19	*n* = 2
3.5	18.99 ± 4.95	20 ± 5.7	22.26 ± 4.16	16.5 ± 6.36
	*n* = 120	*n* = 10	*n* = 19	*n* = 2
4	23.04 ± 6.43	25 ± 7.04	26.63 ± 6.37	18.5 ± 6.36
	*n* = 118	*n* = 10	*n* = 19	*n* = 2
5	22.08 ± 6.4	24.4 ± 6.88	25.74 ± 6.44	18.5 ± 6.36
	*n* = 114	*n* = 10	*n* = 19	*n* = 2
6	20.55 ± 6.27	23.4 ± 6.79	24.05 ± 7.34	18.5 ± 6.36
	*n* = 106	*n* = 10	*n* = 19	*n* = 2
7	18.87 ± 6.71	21.22 ± 6.89	21.05 ± 7.31	16 ± 5.66
	*n* = 100	*n* = 9	*n* = 19	*n* = 2
8	17 ± 7.05	19.86 ± 6.77	18.5 ± 6.82	16 ± 5.66
	*n* = 95	*n* = 7	*n* = 18	*n* = 2
9	14.54 ± 7.02	18.57 ± 5.8	16.71 ± 7.22	15 ± 7.07
	*n* = 81	*n* = 7	*n* = 17	*n* = 2
10	13.06 ± 7.36	15.8 ± 7.09	14.13 ± 8.13	13.5 ± 7.78
	*n* = 70	*n* = 5	*n* = 16	*n* = 2
11	11.43 ± 7.36	15 ± 7.55	13.2 ± 8.78	6
	*n* = 63	*n* = 5	*n* = 15	*n* = 1
12	9.71 ± 7.7	13.25 ± 10.37	12 ± 9.91	4
	*n* = 57	*n* = 4	*n* = 14	*n* = 1

### Associations

A total of 152 boys with DMD were investigated in this study, considering their compliance with medical and rehabilitation therapies. Overall, 91.1% of the patients had a history of corticosteroid use (all types of steroids and non-specified), and 2.7% had a remote history of corticosteroid use. Deflazacort (0.9 mg/kg/d, once per day) was the most commonly used type of corticosteroid in 83.8% of the patients; also, 2% of these patients had a remote history of deflazacort use. Rehabilitation was another standard therapy used in this study. Based on the results, 54.1% of the patients showed compliance with rehabilitation, without any significant difference between the genotype groups. There was no significant difference in terms of disease progression based on the NSAA score between two groups of patients undergoing rehabilitation or not.

## Discussion

In this study, the motor function and ambulatory status, diagnostic methods, and genetic profile of 152 DMD patients were retrospectively analyzed in Iran. The analysis of natural history of the disease showed about 12 months of diagnostic delay in Iran. The majority of DMD patients in the current study reported the time of disease onset to be 4.04 years, whereas the age of confirmed diagnosis was 5.05 years based on the molecular study. Studies conducted in other countries have also demonstrated a long delay in DMD diagnosis [3, 9, 26]. In this regard, Wang et al. reported the age of disease onset to be 3 years; DMD was diagnosed between six and 8 years of age. In a study by Magri et al., the mean delay between the disease onset and DMD diagnosis was 4.9 ± 5.9 years (range: 6 months to 26 years) [9, 26]. The most important causes of this delay were the parents’ delay in reporting the first noticeable symptoms, delayed visit to a doctor, and delayed referral to a medical center.

In several studies, it has been recommended to raise public awareness of the initial symptoms of DMD. In the initial molecular study, it was time-consuming for us to perform further evaluations or secondary genetic studies in patients with negative results. In the present study, motor delay was reported by the parents in 47.7% of cases, which is compatible with previous studies [24, 26]. The LoA occurred in 42.7% of the patients, and the mean age of LoA and wheelchair dependence was 10.9 years; this finding is consistent with some previous reports [3, 8, 9]. On the other hand, in the UK North Star database and other studies, the median LoA age was 13 years [14, 17]. Contracture was reported in 38.9% of cases, and progressive weakness was found in 86.2% of cases.

In the current study, some important issues regarding the progression patterns of disease and the effect of age on disease progression were examined using the NSAA scale. To characterize the disease based on the NSAA, the course of DMD was reported in 152 Iranian boys. The slope of disease progression was analyzed after 4 years of age when the motor function started to decline. The significantly higher NSAA scores of ambulant boys versus the lower NSAA scores of wheelchair boys suggests DMD progression after 4 years. Overall, the NSAA score plays a role in predicting the LoA in DMD patients [14, 17]. With an average NSAA score of 24 at the age of 4 years, the all-inclusive rate of decline in the NSAA scale was 1.67 units/year; this finding was consistent with the parents’ reports of the patients’ age of disease onset in our study. On the other hand, regarding the age at which the maximum NSAA score was reported, previous studies reported older age (6–7 years); however, the maximum NSAA score was close to our report (raw score of 26). It should be noted that in these two studies, the LoA occurred at the age of 13 years, while the LoA age was 10.9 years in our study [14, 17]. Therefore, the NSAA can accurately support clinical trials reporting outcome measures in the ambulant DMD population. It also simplifies the interpretation of possible outcomes in ambulant DMD boys, who may lose ambulation during the study. We hope to provide more information about the motor function and natural history of DMD in Iranian patients that can be helpful in designing improved DMD therapeutic trials.

Additionally, the present study investigated the mutational spectrum of Iranian DMD patients. The distribution and frequency of mutations in this study were similar to the results of other studies on DMD patients [6, 13, 19]. Exon 44 was the most frequently deleted single exon in the present study, while other studies reported exons 45 and 51. Among multiexon deletions in this study, exons 45–50 and exons 45–52 were the most commonly involved exons, which is consistent with the results of previous studies [29, 30]. The largest deletion found in our study extended from exon 3 to exon 44 in the proximal part, while the largest deletion in previous studies was found in exons 8–47. Analysis of the distribution of mutation sites in 79 exons revealed that 47.4% of them were in the distal part, 35.5% were in the proximal part, and 1.3% were in both distal and proximal parts. Distal deletions constituted 57% of deletions in DMD, and their distribution was compatible with previous reports ([24, 30], and [29]).

Mutations in *DMD* gene can lead to a wide spectrum of clinical phenotypes, which are strongly correlated with the characteristics of molecular changes and the degree of dystrophin protein deficiency due to mutations [12, 28]. The genotype-phenotype correlation was characterized in the current study, and the possible effect of DMD genotype on disease progression was analyzed. The progression of disease was examined based on the NSAA score with regard to mutations while considering genetic therapies. Comparison of the NSAA scale in the first years of assessment, before the decline in motor function, indicated significant findings. There was a significant difference in the NSAA score between the deletion and nonsense groups at the age of 3 years (*P* = 0.04), which indicates the better motor function of the nonsense mutation group before disease progression. Based on the results, the status of individuals with duplications deteriorated at a minimally slower rate compared to those with nonsense and deletion mutations. However, this finding is not as significant as the finding of a study by Muntoni F. et al.; it also contradicted the results of a research group analysis by Bello L. [14].

Moreover, no significant difference was found in the age of LoA between the classified genetic groups. Similarly, no significant correlation was observed between the genotypes and motor delay and the rate of LoA. On the other hand, there was a significant association between contracture and mutation type; in other words, contracture was more frequent in the nonsense mutation group compared to other mutation groups. In the present study, no significant correlation was found between the mutation site and phenotypes in Iranian boys, which contradicts the results of a study in South India, reporting the earlier onset of wheelchair dependence in patients with distal mutations [12, 17].

The present results provided information regarding DMD treatment in Iran. Currently, glucocorticoids are the only medications that can definitely improve muscle strength and function in DMD patients, and evidence-based studies have confirmed their efficacy [10]. The rate of steroid treatment has reached 41 to 76% in developed countries, and evidence suggests escalated corticosteroid administration in recent years [18]. However, there are only few studies regarding the efficacy of glucocorticoid therapy. In our database, based on the patients’ medical records (local and referral hospitals), 91.9% of diagnosed DMD patients had a history of glucocorticoid use. Deflazacort was the most commonly used type of corticosteroid therapy (83.8%). The main reason for not using glucocorticoids was the parents’ refusal or steroid side effects. In this study, the medication compliance of each patient was ensured. Overall, compliance with rehabilitation therapy was reported in 54.1% of Iranian DMD boys.

Additionally, the effects of glucocorticoid use and rehabilitation on the natural history of DMD were investigated in this study. Our results related to standard DMD care showed that most cases had a positive history of steroid use and rehabilitation compliance, although there was no significant difference in the application of standard therapies between the mutation groups. Therefore, we could not evaluate the confounding effects of glucocorticoids and rehabilitation on the genotype-phenotype correlation; this limitation can be addressed in future studies. Besides, in this observational study, all available medical records of the patients were evaluated in a five-year census. Another limitation of this study was the difference in the number of patients in the mutation groups that needs to be resolved in future multicentral research.

## Conclusion

The present study examined the phenotypes and mutational characteristics of DMD patients to provide further information on the natural history of DMD, motor function, disease progression, and diagnosis in these patients. Moreover, the management status of DMD was investigated in Iran. The collected findings can promote the development of clinical trials and future molecular therapies in Iran.

## Supplementary Information

**Additional file 1.**

**Additional file 2.**

## Data Availability

The datasets generated and/or analyzed in the current study are available in the CLINVAR repository (accession numbers: SCV002102482, SCV002102475, SCV002102483, SCV002102474, and SCV002102481) (persistent link: https://www.ncbi.nlm.nih.gov/clinvar/submitters/506651/). The raw data must be analyzed, and the negative or uninformative datasets must be summarized; however, they are available from the corresponding author on reasonable request. All clinical and genomic data and materials are available from the corresponding author upon the reviewers’ request.
