# Supplementary material for: Characteristics of disease progression and genetic correlation in ambulatory Iranian boys with Duchenne muscular dystrophy
Source: BMC Neurol. 2022 May 2;22:162. doi: 10.1186/s12883-022-02687-1 (PMC9059913; doi:10.1186/s12883-022-02687-1)
Supplement: Supplementary file 1 — Additional file 1. [file 12883_2022_2687_MOESM1_ESM.docx]

| **MLPA** | | **NGS** | | |  |
| --- | --- | --- | --- | --- | --- |
| **Mutation** | **Exon number** | **Mutation (Common Name)** | **International Name** | **Clinical Significance** | **Number of Reports/Clinvar Submission** |
| **Deletion**  **Duplication** | 11, (12-16), (18-21), 19,  (19-23), (2-7), (23-26), (3-11)  , (3-26), (3-42), (3-44), (3-7), (3-9), (3-30), (3-43), 45,  (35-43), (37-43), (39-62), (41-43), (43-50), 44, (44-48),  (44-51), 45, (45-47)*, (45-49),  (45-50), 52, (45-53), 46, (46-47), (46-48), (46-50), (46-51), (46-52), (46-53), (46-55),  (46-62), 47, (47-48), (48-50),  (48-52), (49-50), (49-52),  (5-29), 50, (50-52), (50-54), 51,  (51-53), 52, 53, (53-55), 55, (56-62), 6, (6 -41), 7, 70, 8, (8-12), (8-44),  (8-15), (8-17), 9, 2,  exon1-promoter region  (3,4,6,8,12,13,16,17,19,41-45)  2, (3-9), (3-11), (3-26), 8, 9, (12-16), 50 | Nonsense | NM_004006.2:c.2521C>T p.(Gln841*) Exon 20 | Pathogenic | 5 |
|  |  | Nonsense | NM_004006.2:c.2032C>T p.(Gln678*) Exon 17 | Pathogenic | 6 |
|  |  | Nonsense | NM_004006.2:c.6970C>T p.Gln2264X Exon 47 | Pathogenic | 2 |
|  |  | Nonsense | NM_004006.2:c.5899C>T p.(Arg1967*) Exon 41 | Pathogenic | 48 |
|  |  | Nonsense | NM_004006.2:c.10477C>T (p.Gln3493*) Exon 74 | Pathogenic | 5 |
|  |  | Nonsense | NM_004006.2:c.5114C>A p.(Ser1705*) Exon 9 | Likely pathogenic | Not Reported/ SUB11157901 |
|  |  | Nonsense | NM_004006.2:c.358G>T p.(Glu1120Ter) Exon 25 | Pathogenic | 1 |
|  |  | Nonsense | NM_004006.2:c.6139C>T p.Gln2047Ter Exon 43 | pathogenic | 1 |
|  |  | Nonsense | NM_004006.2:c.3414G>A p.(Trp1138*) Exon 25 | pathogenic | 4 |
|  |  | Nonsense | NM_004006.2:c.10108C>T p.(Arg3370*) Exon 70 | Pathogenic | 59 |
|  |  | Nonsense | NM_004006.2:c.3414G>A p.(Trp1138*) Exon 25 | pathogenic | 4 |
|  |  | Nonsense | NM_004006.2:c.3210del p.Leu1071* Exon 24 | Likely pathogenic | Not Reported/ SUB11157923 |
|  |  | Nonsense | NM_004006.2:c.2479G>T p(Glu827Ter) Exon 20 | Pathogenic | 3 |
|  |  | Nonsense | NM_004006.2:c.2479G>T p.(Glu827Ter) Exon 20 | Pathogenic | 3 |
|  |  | Nonsense | NM_004006.2:c.8608C>T (p.R2870X) Exon 58 | Pathogenic | 56 |
|  |  | Nonsense | NM_004006.2:c.4545_4549del p.(Lys1516*) Exon 33 | Pathogenic | 8 |
|  |  | Nonsense | NM_004006.2:c.3595G>T (p.Glu1199*) Exon 26 | Pathogenic | 3 |
|  |  | Nonsense | NM_004006.2:c.3414G>A p.(Trp1138*) Exon 25 | Likely pathogenic | 4 |
|  |  | Splice site | NM_004006.2:c.9564-2del Exon 66 | Likely pathogenic | 1 |
|  |  | Splice site | NM_004006.2:c.93+1G>A Intron 2 | Pathogenic | 1 |
|  |  | Deletion  (frameshift) | NM_004006.2:c.2416_2417delGA p.Glu806ThrfsX15  Exon 20 | Likely pathogenic | Not Reported/ SUB11158031 |
|  |  | Deletion  (frameshift) | NM_004006.2:c.287del p.(Ser96llefs*5)  Exon 3 | Likely pathogenic | Not Reported/ SUB11158042 |
|  |  | Deletion  (frameshift) | NM_004006.2:c.1402_1403del p.(Glu468Argfs*14)  Exon 12 | Likely pathogenic | Not Reported/ SUB11158045 |
|  |  | Deletion  (frameshift) | NM_004006.2:c.1083_1090del p.(Leu362Thrfs*6)  Exon 10 | Likely Pathogenic | 1 |
|  |  | Deletion  (frameshift) | NM_004006.2:c.5697delA(p.Lys1899Asnfs*2)  Exon 40 | Pathogenic | 6 |

**Supplementary Table S1.** The variants summary, pathogenicity and number of reports for each variant are categorized according to the Global Variome shared LOVD database (Supplementary Table S1). * In terms of clinical feature, it is consistent with DMD and in order to differentiate it from BMD, it is suggested that functional studies be performed.
