# Supplementary material for: Characteristics of disease progression and genetic correlation in ambulatory Iranian boys with Duchenne muscular dystrophy
Source: BMC Neurol. 2022 May 2;22:162. doi: 10.1186/s12883-022-02687-1 (PMC9059913; doi:10.1186/s12883-022-02687-1)
Supplement: Supplementary file 2 — Additional file 2. [file 12883_2022_2687_MOESM2_ESM.docx]

| **phenotype**  **Genotype** | Del 3-7 exon  N=1 | Del 45  N=4 | Del 52  N=4 | P value |
| --- | --- | --- | --- | --- |
| Wheelchair dependency | 1/1(100%) | 0/4(0%) | 2/4(50%) | 0.20 |
| Contracture | 0/1(0%) | 2/4(50%) | 1/4(25%) | 0.90 |
| Progressive weakness | 1/1(100%) | 4/4(100%) | 4/4(100%) | 1.00 |
| LOA age(Mean) | 11 years | 10 years | 10.5 years | 0.97 |
| Disease onset age(Mean) | 3 years | 2.7 years | 3 years | 0.44 |
| Motor delay | 1/1100%) | 2/4(50%) | ¾(75%) | 0.49 |
| Compliance with rehabilitation | 1/1(100%) | 1/4(25%) | 2/4(50%) | 0.60 |
| Steroid taking | 1/1(100%) | 4/4(100%) | 4/4(100%) | 1.00 |
| NSAA score-3 years | 4.00±2.11 | 10.25±1.70 | 11.25±2.50 | 0.03 |
| NSAA score-3.5 years | 7.00±3.02 | 16.00±3.16 | 19.00±5.09 | 0.33 |
| NSAA score-4 years | 8.00±2.11 | 19.25±2.75 | 22.75±6.18 | 0.47 |
| NSAA score-5 years | 8.00±1.20 | 19.25±2.75 | 20.00±4.39 | 0.06 |
| NSAA score-6 years | 8.00±3.43 | 18.25±2.62 | 17.50±2.88 | 0.08 |
| NSAA score-7 years | 8.00±3.90 | 17.00±2.64 | 15.00±4.08 | 0.16 |
| NSAA score-8 years | 8.00±3.23 | 16.33±1.52 | 13.66±5.13 | 0.60 |
| NSAA score-9 years | 8.00±3.23 | 16.50±2.12 | 12.00±5.29 | 0.62 |
| NSAA score-10 years | 6.00±4.03 | 16.50±2.12 | 10.33±6.80 | 0.58 |
| NSAA score-11 years | 4.00±2.01 | 18.00±4.01 | 9.00±7.81 | 0.53 |
| NSAA score-12 years | 4.00±2.01 | 18.00±4.01 | 8.33±8.38 | 0.66 |

**Supplementary Table S2.** Genotype-phenotype correlation in the deletion mutation subgroups.
